# Whole-blood model reveals granulocytes as key sites of dengue virus propagation, expanding understanding of disease pathogenesis

**DOI:** 10.1128/mbio.01505-24

**Published:** 2024-11-14

**Authors:** Hansa Praneechit, Somchai Thiemmeca, Dararat Prayongkul, Kessiri Kongmanas, Dumrong Mairiang, Nuntaya Punyadee, Adisak Songjaeng, Nattaya Tangthawornchaikul, Nasikarn Angkasekwinai, Kanokwan Sriruksa, Yupin Suputtamongkol, Wannee Limpitikul, John P. Atkinson, Panisadee Avirutnan

**Affiliations:** 1 Graduate Program in Immunology, Department of Immunology, Faculty of Medicine Siriraj Hospital, Mahidol University, Bangkok, Thailand; 2 Division of Dengue Hemorrhagic Fever Research, Research Department, Faculty of Medicine Siriraj Hospital, Mahidol University, Bangkok, Thailand; 3 Siriraj Center of Research Excellence in Dengue and Emerging Pathogens, Faculty of Medicine Siriraj Hospital, Mahidol University, Bangkok, Thailand; 4 Molecular Biology of Dengue and Flaviviruses Research Team, Medical Molecular Biotechnology Research Group, National Center for Genetic Engineering and Biotechnology (BIOTEC), National Science and Technology Development Agency (NSTDA), Bangkok, Thailand; 5 Department of Medicine, Faculty of Medicine Siriraj Hospital, Mahidol University, Bangkok, Thailand; 6 Pediatric Department, Khon Kaen Hospital, Ministry of Public Health, Khon Kaen, Thailand; 7 Pediatric Department, Songkhla Hospital, Ministry of Public Health, Songkhla, Thailand; 8 Division of Rheumatology, Department of Medicine, Washington University School of Medicine, St. Louis, Missouri, USA; Catholic University of America, Washington, DC, USA; Boston Children's Hospital, Boston, Massachusetts, USA

**Keywords:** neutrophils, *in vitro* whole-blood infection model, dengue virus, granulocytes, dengue target cells

## Abstract

Dengue virus (DENV) infection poses a significant global health threat, yet our understanding of its immunopathogenesis remains incomplete due to limitations of existing models. Here, we establish an *in vitro* whole-blood model using hirudin, an anticoagulant that preserves complement activity and cellular interactions, to study DENV infection. Our model reveals the susceptibility of all major leukocyte populations to DENV infection, with monocytes and granulocytes demonstrating high permissiveness and production of infectious virus progeny. Notably, granulocytes emerge as previously unrecognized targets of DENV infection, highlighting the importance of studying viral tropism within a physiologically relevant context. We also observed efficient DENV binding to B cells, but limited production of infectious virus, suggesting a potential role in viral sequestration or immune dysregulation. Interestingly, both NK and T cells, while less permissive, were also found to be susceptible to DENV infection. Our *ex vivo* analysis of whole blood from DENV-infected patients confirms the susceptibility of granulocytes, monocytes, B cells, natural killer cells, and T cells to infection, further validating the clinical relevance of our model. Additionally, we observed dynamic changes in circulating blood cell populations during acute dengue, potentially reflecting both direct virus-mediated effects and immune responses. This whole-blood model offers a valuable tool for investigating the complex interplay between DENV and host factors, facilitating a deeper understanding of dengue pathogenesis and ultimately contributing to the development of novel therapeutic strategies.

**IMPORTANCE:**

Dengue virus (DENV) infection is a significant global health threat, with increasing incidence in endemic regions and expanding geographic range due to factors like global warming. Current models for studying DENV pathogenesis often lack the complexity of the human immune system, hindering the development of effective therapies and vaccines. To address this, we have established the first *in vitro* whole-blood model using hirudin, preserving critical immune components and cellular interactions. This model reveals granulocytes as previously unrecognized targets of productive DENV infection, challenging existing paradigms of viral tropism. Our *ex vivo* analysis of patient blood samples confirms the clinical relevance of this finding and validates our model’s utility. This unique model offers a powerful platform for future studies to dissect the complex interactions between DENV and the host immune system, including the roles of different leukocyte populations, ultimately informing the development of novel therapeutic strategies to combat this devastating disease.

## INTRODUCTION

Dengue virus (DENV) poses a significant global health threat, with approximately 390 million infections and 10,000 deaths annually across over 125 countries (1, 2). Factors, such as urbanization, migration, and global warming, have contributed to a dramatic increase in dengue cases over the past three decades (3). DENV, an enveloped virus with a positive-sense single-stranded RNA genome, exists in four serotypes (DENV1 to DENV4) and is transmitted to humans through infected *Aedes* mosquitoes (4). Of particular concern are antibody-dependent enhancement (ADE), where non-neutralizing antibodies from a previous DENV infection can facilitate infection of monocytes and other Fc-gamma receptor-bearing cells, and “original antigenic sin,“ where the immune response to a secondary infection is biased toward the first encountered serotype (5–7). These phenomena complicate the immune response and can contribute to more severe disease.

The damaging effects of dengue illness stem from the dysregulated interplay between DENV and the host’s immune response. This includes massive complement activation (8–11) and the release of excessive pro-inflammatory cytokines, known as a “cytokine storm“ (12). The similarity between serotypes leads to cross-reactivity, where antibodies and memory cells from a previous infection can worsen a secondary infection with a different serotype (13). This cross-reactivity is a key factor in both ADE and original antigenic sin, leading to the production of cross-reactive, non-neutralizing antibodies that can enhance infection and exaggerated inflammatory response (6, 7, 14).

Neutrophils, the most abundant leukocytes in the blood, serve as a critical first line of defense against pathogens (15–17). In DENV infection, evidence suggests neutrophils play a multifaceted and potentially harmful role (18, 19). They become highly activated, releasing reactive oxygen species and neutrophil extracellular traps (NETs), which can contribute to both viral clearance and tissue damage (20, 21). Research indicates that specific neutrophil subsets, such as those with low CD62L and high CD16 expression or immature neutrophils, may be particularly associated with severe dengue (22). Neutrophils also interact closely with other immune cells and serum proteins, potentially amplifying the inflammatory response (23, 24). However, the precise mechanisms underlying neutrophil activity in dengue pathogenesis remain unclear due to limitations in existing *in vitro* models, which often do not fully replicate the interactions of neutrophils with other circulating cells or serum components. Additionally, isolated neutrophils have a short lifespan in culture, further complicating their study (25).

The concept of using whole-blood models to study host–pathogen interactions was pioneered in the field of bacterial infection research (26, 27). The use of hirudin, which preserves complement function unlike heparin and ethylenediaminetetraacetic acid (EDTA), was crucial for elucidating complex immune responses during bacterial infections (26). This approach allowed researchers to uncover details of monocyte and neutrophil interplay, as well as novel bacterial immune evasion mechanisms (28, 29). Building on these successes, we propose an *in vitro* whole blood model using hirudin to explore DENV infection. This model will preserve the presence of neutrophils, other key immune cell types, and serum proteins including complement, mimicking natural infection in human blood. In this study, we investigated DENV tropism across different blood cell populations to gain a deeper understanding of how the interplay between DENV, the diverse cellular components of blood, and serum proteins influences dengue pathogenesis. Additionally, we directly compared the results of DENV-positive cell populations from our *in vitro* whole-blood model with fresh *ex vivo* whole-blood samples from adult and pediatric dengue patients. This comprehensive approach will shed light on DENV burden within the bloodstream, potentially influencing viral dissemination to other susceptible organs like the liver, spleen, and lymph nodes.

## RESULTS

### Compatibility of hirudin to whole-blood infection model for DENV infection

**Fig 1:**
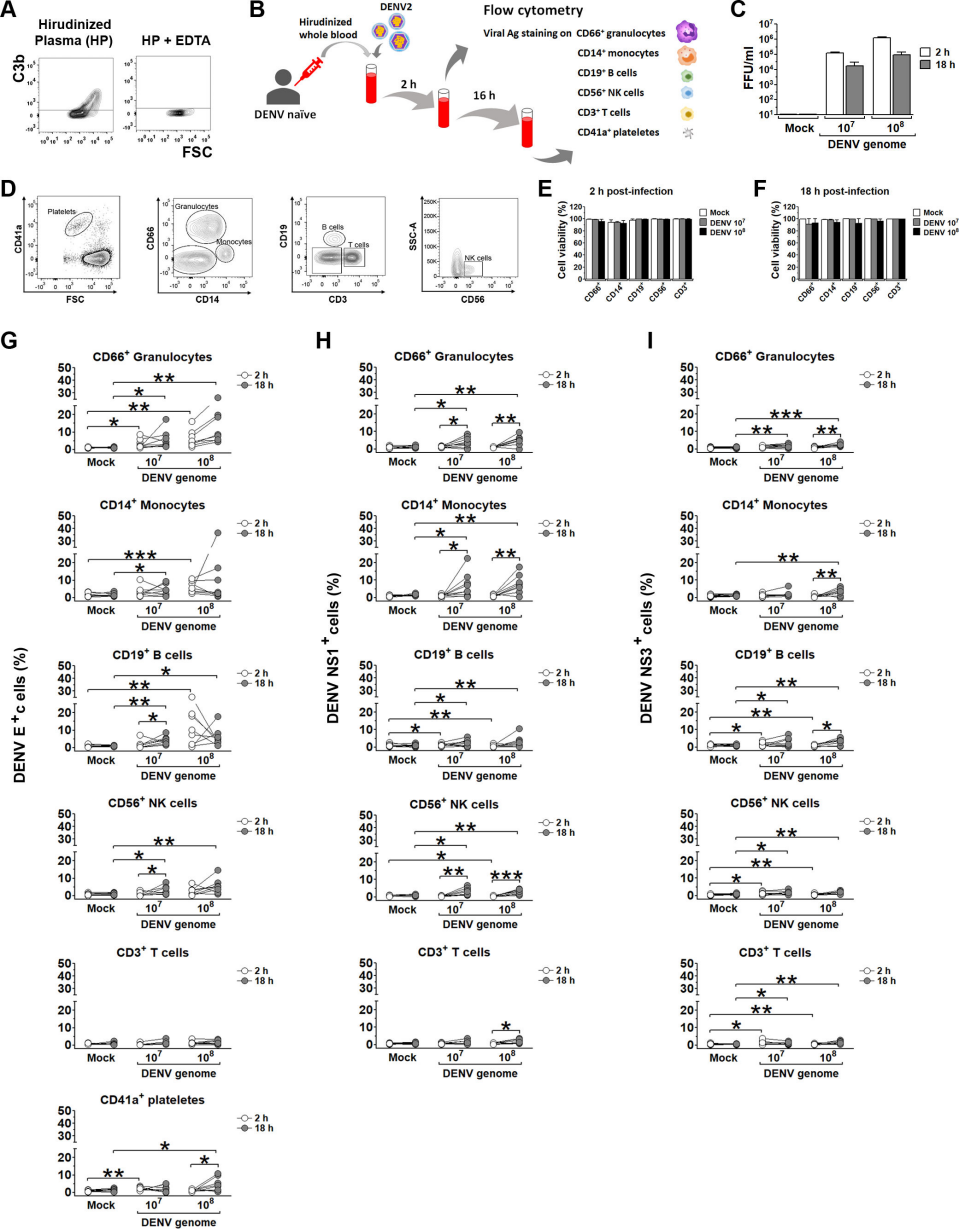
Hirudin compatibility with a whole-blood DENV infection model. (**A**) Hirudin preserves complement function. Plasma was separated from hirudinized whole blood or EDTA-treated hirudinized blood after 2 h, then incubated with yeast cells. C3b deposition on yeast was detected by flow cytometry. (**B**) Diagram of the *in vitro* whole-blood DENV infection model (18 h post-infection). At 2 and 18 h post-infection, each cell population was analyzed based on their size, granularity, and CD markers for cell viability and dengue antigens. (**C**) Hirudin does not interfere with DENV infectivity. Hirudinized blood from DENV-naïve donors was incubated with mock or DENV (10⁷ or 10⁸ genome copies/mL). Infectious DENV titers in plasma were determined by focus-forming unit (FFU) assay at 2 h (white bars) and 18 h (gray bars) post-infection. (**D**) Representative gating strategy for analyzing DENV-infected whole blood. Cells were gated based on forward scatter (FSC), side scatter (SSC), and established CD markers to distinguish platelets (CD41a^+^), granulocytes (CD66^+^), monocytes (CD14^+^), B (CD19^+^) and T (CD3^+^) lymphocytes, and NK cells (CD56^+^). (**E, F**) Hirudin maintains leukocyte viability in a whole-blood infection model. Cell viability in each white blood cell (WBC) population was determined by live/dead staining and flow cytometry after incubation with mock (white bars), DENV 10⁷ genome copies/mL (gray bars), or 10⁸ genome copies/mL (black bars) at 2 h (**E**) and 18 h (**F**) post-infection. (**G–I**) DENV infection in each cell population in a whole-blood infection model. Percentages of DENV-positive cells, as determined by surface DENV envelope (**E**), protein (**G**), intracellular NS1 (**H**), and intracellular NS3 (**I**) in each WBC cell population were determined at 2 h (white circles) and 18 h (gray circles) post-infection. Data are presented as individual dot plots from 8 to 10 independent experiments. Student’s *t*-test was used to compare expression levels of DENV antigens among each time-point and each condition in each cell population. Asterisks (*, **, and ***) indicate statistical significance (*P* < 0.05, *P* < 0.01, and *P* < 0.005, respectively).

In contrast to heparin and EDTA, hirudin maintains blood complement function (30). To confirm this, we measured complement activation in hirudinized plasma by detection of C3b on yeast cells. C3b deposition occurred in the presence of hirudin, but not after EDTA treatment (Fig. 1A). Next, we assessed DENV infectivity and cell viability in our *in vitro* whole-blood model using hirudinized blood. Blood was incubated with DENV2 strain 16681 at clinically relevant concentrations (10^7^ or 10^8^ genome copies) (31) in the *in vitro* model (Fig. 1B). Platelets, granulocytes, monocytes, B cells, T cells, and natural killer (NK) cells were identified by their characteristic forward scatter (FSC), side scatter (SSC), and surface marker expression (Fig. 1B and D). Infectious DENV was detectable in plasma at 2 h but showed a slight decrease by 18 h post-infection (Fig. 1C). This reduction is likely due to a combination of factors, including the binding and internalization of the virus into whole-blood cells, as well as the relatively low level of viral progeny production at this early time point (32–34). Leukocyte viability remained high (>90%) across all populations at both 2 and 18 h post-infection, regardless of DENV dose (Fig. 1E and F). These results indicate that hirudin preserves serum complement activity, does not affect infectivity of DENV, and maintains the viability of all major leukocyte populations through the initial 18 h of infection, covering the first round of DENV replication (32–34).

Next, we examined DENV infection in circulating cells by detecting viral proteins. DENV envelope protein (E), a structural component of virions, indicates initial viral binding and subsequent replication (33). At 2 h post-infection, E was predominantly detected on CD66^+^ granulocytes, CD14^+^ monocytes, and CD19^+^ B cells, with lower levels in CD56^+^ NK cells, CD3^+^ T cells, and CD41a^+^ platelets, in a dose-dependent manner (Fig. 1G). By 18 h, E expression significantly increased in B and NK cells (at 10^7^ genome copies) and platelets (at 10^8^ genome copies), with a trend toward increased expression observed in granulocytes and monocytes, suggesting DENV binding followed by replication in these cell types. Notably, granulocytes exhibited consistently high E expression, while T cells displayed very low levels at both time points (Fig. 1G).

To confirm DENV replication, we also detected NS1 and NS3, which are components of viral replication complexes (12). At 2 h, NS1 and NS3 were largely undetectable in granulocytes and monocytes, with rare detection in lymphocytes (Fig. 1H and I). By 18 h, both proteins were frequently detected in granulocytes, monocytes, lymphocytes, and NK cells, indicating DENV replication in those cells, with NS1 exceeding NS3 in monocytes, granulocytes, and NK cells, potentially due to phagocytosis of secreted NS1 (Fig. 1H and I). While T cells showed low susceptibility to DENV, with minimal E detection (Fig. 1G), a small proportion of cells expressed NS1 and NS3, suggesting limited replication (Fig. 1H and I). Interestingly, B cells displayed high initial E levels but low NS1/NS3 expression (Fig. 1G through I), indicating susceptibility to binding but potentially limited replication. Due to technical limitations, intracellular NS1/NS3 staining could not be performed in platelets. Overall, these findings demonstrate that hirudin is a suitable anticoagulant for *in vitro* whole-blood models of DENV infection, as it maintains complement activity, viral infectivity, and cell viability. Notably, we observed significant DENV infection of granulocytes, suggesting a potential role for these cells in viral replication.

### Granulocytes as novel DENV target cells supporting productive DENV infection

**Fig 2:**
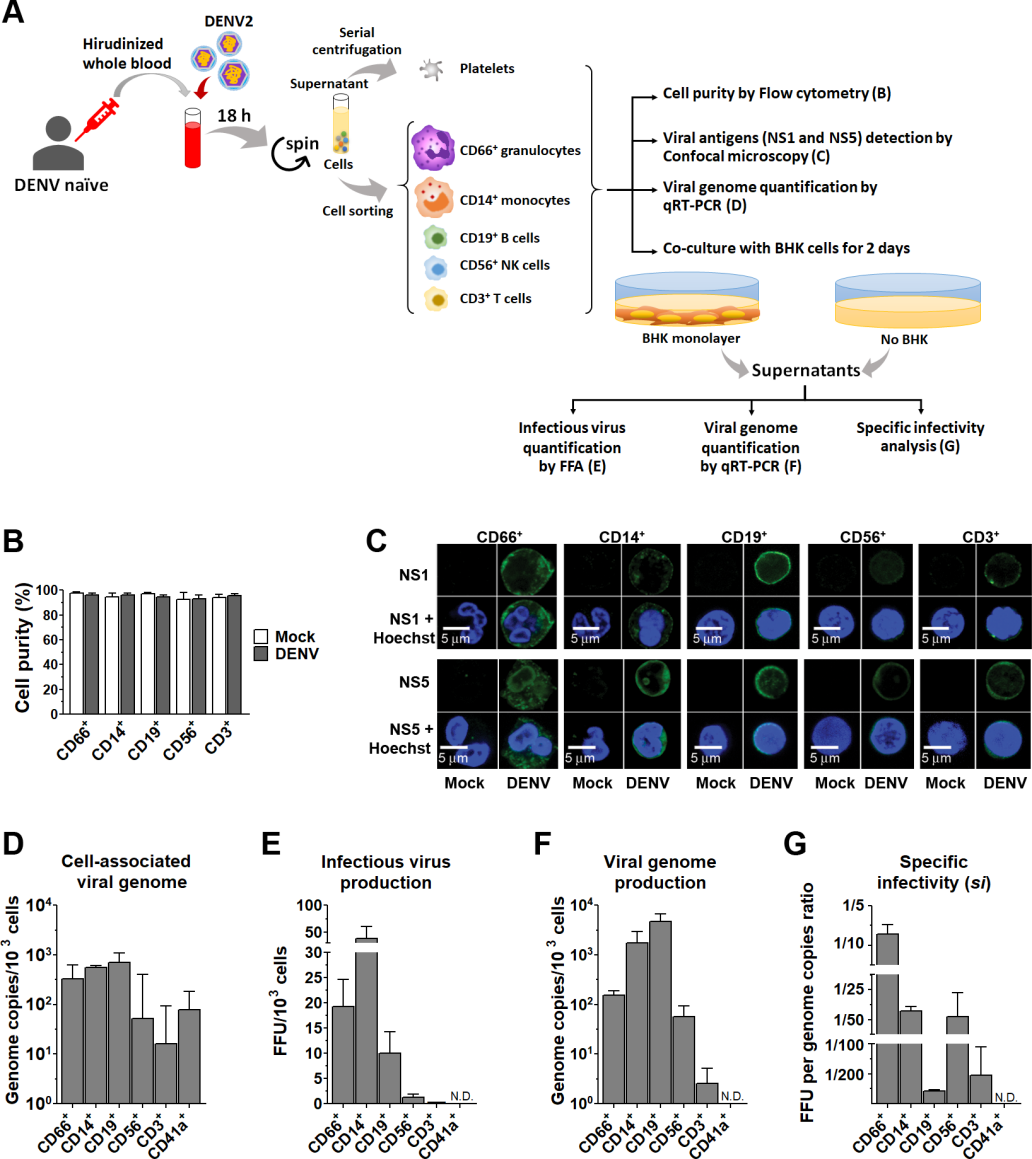
DENV infection and replication efficiency in different blood cell populations. (**A**) Schematic diagram of the experimental setup for analyzing DENV infection and replication in sorted blood cell population 18 h post-infection with DENV at 10⁸ genome copies/mL. (**B**) Purity assessment of each sorted WBC population from mock-infected (white bars) or DENV-infected (black bars) whole blood. (**C**) Confocal microscopy images of DENV-infected WBCs. Intracellular DENV NS1 and NS5 proteins are shown in green, and nuclei are stained with Hoechst dye (blue). (**D**) Quantification of cell-associated DENV RNA in each sorted cell population by qRT-PCR. (**E**) Infectious DENV titers (FFU/10³ cells) in supernatants from co-cultures of sorted blood cell populations with permissive BHK cells for 2 days. (**F**) DENV genome copies (per 10³ cells) in supernatants from co-cultures as in panel **E**. (**G**) Specific infectivity (*si*) of DENV in each cell population, calculated as the ratio of infectious DENV titers (FFU) to viral genome copies. Data are representative of three independent experiments. N.D. indicates "not detected."

To investigate the efficiency of DENV infection and the generation of infectious virions from each cell type in our whole-blood infection model, we isolated all cell populations at 18 h post-infection and subsequently examined the susceptibility of each cell type to DENV infection. This analysis included cell-associated viral antigens and genome, as well as the potential for virus progeny production, as illustrated in the experimental schematic in Fig. 2A. Cell purity of each white blood cell (WBC) population was confirmed to be >90% (Fig. 2B). The purity of isolated platelets was >98% after determined by staining with platelet marker CD41a, leukocyte marker CD45, and red blood cell marker CD235a (data not shown). Cells were then subjected to confocal microscopy to confirm DENV replication by the presence of NS1 and the viral RNA polymerase NS5 within the cytoplasm (Fig. 2C). We also quantified cell-associated viral genomes by qRT-PCR (Fig. 2D). Strong intracellular NS1 and NS5 expression in phagocytic CD66^+^ granulocytes and CD14^+^ monocytes and CD19^+^ B cells confirmed DENV replication in these cell types (Fig. 2C). Weaker fluorescence signals were observed in CD56^+^ NK cells and CD3^+^ T cells, likely due to smaller cytoplasmic volume (Fig. 2C). Quantitative RT-PCR revealed varying levels of cell-associated DENV RNA across leukocyte populations and platelets (Fig. 2D). Notably, CD66^+^ granulocytes exhibited high viral loads (331 genome copies/10^3^ cells), comparable to monocytes (562 genome copies/10^3^ cells) and B cells (708 genome copies/10^3^ cells) (Fig. 2D), suggesting granulocytes as a previously unrecognized target for DENV.

To assess infectious virus production, defined numbers of sorted leukocyte populations were co-cultured with or without susceptible baby hamster kidney (BHK) cells for 2 days. The addition of BHK cells served to amplify any low levels of virus progeny, ensuring detection by focus-forming assay (FFA). Supernatants were then analyzed for the presence of viral genome and infectious activity (Fig. 2A). No detectable DENV was found in cultures without BHK cells (data not shown). This is likely due to a combination of the following factors: the low initial infection rates observed in some leukocyte populations (<20%; Fig. 1), and the potential reduction in cell viability following isolation and extended culture. Additionally, the limited sensitivity of FFA for detecting very low levels of infectious virus may have contributed to this observation. Control experiments with BHK cells alone, cultured in the final washed media from isolated WBCs, yielded no detectable virus (data not shown), confirming that detected viral progeny originated from the blood cells.

In co-cultures with BHK cells, among all cell populations, infectious DENV was effectively produced by monocytes (39 FFU/10^3^ cells) and, notably, granulocytes (19 FFU/10^3^ cells), confirming their role in productive DENV infection. This was followed by B cells (10 FFU/10^3^ cells) (Fig. 2E). NK cells also produced infectious virus, albeit at lower levels, while T cells yielded nearly undetectable amounts (Fig. 2E). Viral RNA production in co-cultures followed a similar pattern, except that the highest viral RNA copies were observed in the supernatant of B cell-BHK co-cultures (Fig. 2F). Notably, despite detectable cell-associated DENV RNA (Fig. 2D), platelets did not produce infectious virus in co-culture (Fig. 2F), consistent with previous findings. This suggests that platelets, despite being susceptible to DENV infection, are not able to produce infectious virus progeny, a phenomenon known as abortive infection (35, 36). Specific infectivity (SI), the ratio of infectious virus to total viral RNA (37), revealed granulocytes as the most efficient producers of infectious DENV (1 FFU/8 genome copies), followed by monocytes and NK cells (1 FFU/39 and 1 FFU/46 genome copies, respectively) (Fig. 2G). T and B cells displayed dramatically lower SI (1 FFU/210 and 1 FFU/467 genome copies, respectively) (Fig. 2G).

Our whole-blood infection model not only confirms monocytes as a primary target for DENV replication but also reveals granulocytes as a previously unrecognized site of productive infection, highlighting the potential of this system to uncover novel aspects of DENV pathogenesis. Surprisingly, despite being highly susceptible to DENV binding and infection, B cells predominantly produced non-infectious virions, exhibiting a lower specific infectivity than NK cells. This suggests that distinct cellular mechanisms may regulate DENV replication efficiency across different leukocyte populations.

### *In vivo* evidence of DENV infection in granulocytes and other leukocytes

**Fig 3:**
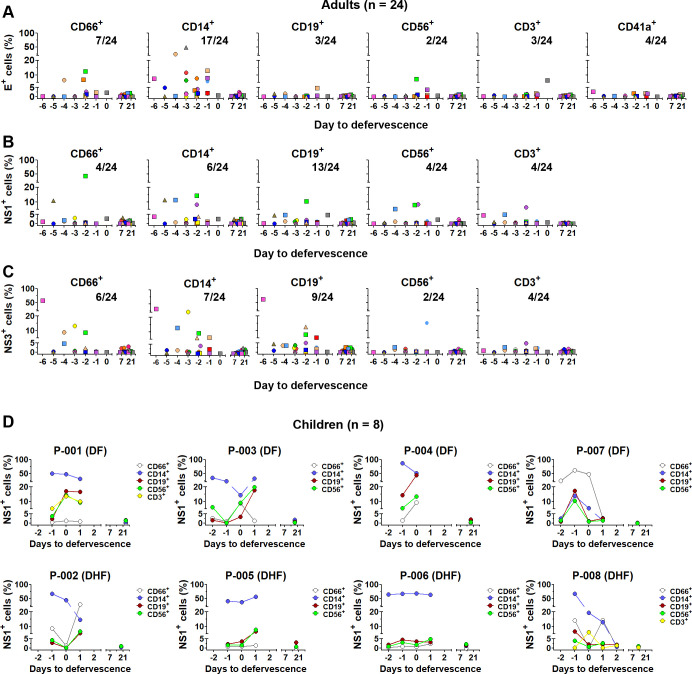
DENV antigen detection in whole blood from adult and pediatric patients. (**A**) Representative gating strategy for flow cytometry analysis of DENV-infected patient whole blood. Cell populations were identified based on forward scatter (FSC), side scatter (SSC), and the following CD markers: platelets (CD41a^+^), granulocytes (CD66^+^), monocytes (CD14^+^), B cells (CD19^+^) and T cells (CD3^+^), and NK cells (CD56^+^). (**B–D**) Percentages of cells positive for surface DENV envelope (**E**), protein (**B**), intracellular NS1 (**C**), or intracellular NS3 (**D**) in the indicated cell populations from adult DENV-infected patients (*n* = 24). Whole blood was collected at the following two time points: acute (1–6 days before defervescence) and convalescent (7–24 days after defervescence). Each symbol represents an individual patient. The *X*/*X* number in each plot indicates the number of patients with detectable positive cells out of the total number of analyzed for that cell population. (**E**) Kinetics of NS1-positive cells in the indicated cell populations from pediatric DENV-infected patients (*n* = 8; four with dengue fever [DF], four with dengue hemorrhagic fever [DHF]). Whole blood was collected daily during the acute phase, with one convalescent sample. Lines represent individual patients.

To validate our *in vitro* findings of DENV infection in granulocytes and assess its clinical relevance, we examined DENV tropism in whole-blood samples from adult and pediatric dengue patients. We analyzed whole blood from 24 DENV-infected adult patients during the acute (1–6 days before defervescence, designated as day 0) and convalescent (7–24 days after defervescence) phases. Platelets, granulocytes, monocytes, B cells, T cells, and NK cells were identified by their characteristic forward scatter (FSC), side scatter (SSC), and surface marker expression, as detailed in our gating strategy (Fig. 3A). Notably, during the febrile phase, a significant proportion of CD66^+^ granulocytes (ranging from 1.8% to 12.5%) were associated with DENV E protein in 7 out of 24 patients, consistent with our *in vitro* observations and confirming that granulocytes are targets of DENV infection in humans (Fig. 3B). Additionally, a high proportion of DENV E-positive cells were also detected in CD14^+^ monocytes (1.5% to 48.7% of cells were positive in 17 out of 24 patients), while smaller proportions were found in lymphocytes (CD19^+^ B cells [1.6%–4.7%, 3/24 patients], CD56^+^ NK cells [3.8%–7.3%, 2/24 patients], CD3^+^ T cells [1.5%–6.5%, 3/24 patients], and CD41a^+^ platelets [1.7%–7.0%, 4/24 patients]) (Fig. 3B).

To confirm active replication of DENV in these cell types, we analyzed intracellular NS1 and NS3 expression, markers of DENV replication complexes (38), in all cell population except platelets. Notably, both NS1 and NS3 were detected across all WBC populations, with the highest proportions of positive cells found in monocytes (NS1: 3.8%–14.2%, 6/24 patients; NS3: 4.2%–32.1%, 7/24 patients), B cells (NS1: 1.2%–10.5%, 13/24 patients; NS3: 1.9%–61.2%, 9/24 patients), and granulocytes (NS1: 2.6%–42.6%, 4/24 patients; NS3: 2.1%–56.8%, 6/24 patients) (Fig. 3C and D). This further validates that granulocytes, like monocytes and B cells, are targets of DENV infection in humans. NK cells (NS1: 2.3%–8.5%, 4/24 patients; NS3: 2.7%–15.1%, 2/24 patients) and T cells (NS1: 1.4%–6.5%, 4/24 patients; NS3: 2.1%–4.9%, 4/24 patients) showed lower proportions of NS1-/NS3-positive cells (Fig. 3C and D). The lower observed frequencies of infected cells in patient samples may be attributed to the later stage of infection (collected 1–6 days into the febrile phase, approximately 6–11 days post-infection) (39, 40) compared to our *in vitro* whole-blood model (18 h post-infection). At this later stage, viremia is typically declining (41).

In a separate cohort of eight pediatric patients (four with dengue fever [DF] and four with dengue hemorrhagic fever [DHF]), we observed a similar pattern of NS1 expression in circulating leukocytes during the acute phase. High proportions of NS1-positive cells were detected in CD14^+^ monocytes (1.5%–86.6%, 8/8 patients), CD19^+^ B lymphocytes (1.6%–43.9%, 8/8 patients), and CD66^+^ granulocytes (1.2%–61.7%, 5/8 patients) (Fig. 3D). Lower proportions of NS1-positive cells were found in CD56^+^ NK cells (1.5%–19.6%, 8/8 patients), while CD3^+^ T cells showed 1.5%–13.6% in two patients with available data (Fig. 3D). The increased percentage of NS1-positive cells and the higher proportion of patients with detectable DENV-positive cells in the pediatric cohort (Fig. 3D) compared to the adults are likely due to the enhanced sensitivity of indirect NS1 staining used in pediatric samples compared to direct NS1 staining in adult samples (Fig. 3B). Importantly, kinetic analysis of the pediatric cohort revealed detectable DENV-positive cells, especially in granulocyte, monocyte, and B-cell populations, even on the day of hospital discharge (1 or 2 days after defervescence) (Fig. 3D). Individual patient data for NS1-positive cells in each population varied considerably in magnitude and showed distinct kinetic patterns (Fig. 3D). As expected, all DENV antigens tested were negative in all cell types in whole-blood samples obtained during the convalescent phase, confirming the specificity of our findings for acute DENV infection.

To investigate the relationship between the extent of viral infection in blood cells and the overall viral burden in the patient, we analyzed the correlation of NS1/NS3 detection efficiency with plasma viral loads from 24 adult and the first time-point in six out of eight pediatric patients (data for viremia was unavailable for the remaining two pediatric patients). Notably, no significant correlation was observed, as shown in Fig. S1 and S2. This suggests that factors beyond viremia levels, such as immune responses or cellular permissiveness, might play a crucial role in determining the extent of DENV infection in different blood cell populations. It is also important to consider that the timing of sample collection, several days after the initial infection, may influence this observed lack of correlation. In a controlled human infection study, where blood specimens can be obtained at earlier time points post-infection, a different relationship between viremia and the frequency of antigen-positive cells might be observed.

#### Impact of DENV infection on circulating blood cell populations

**Fig 4:**
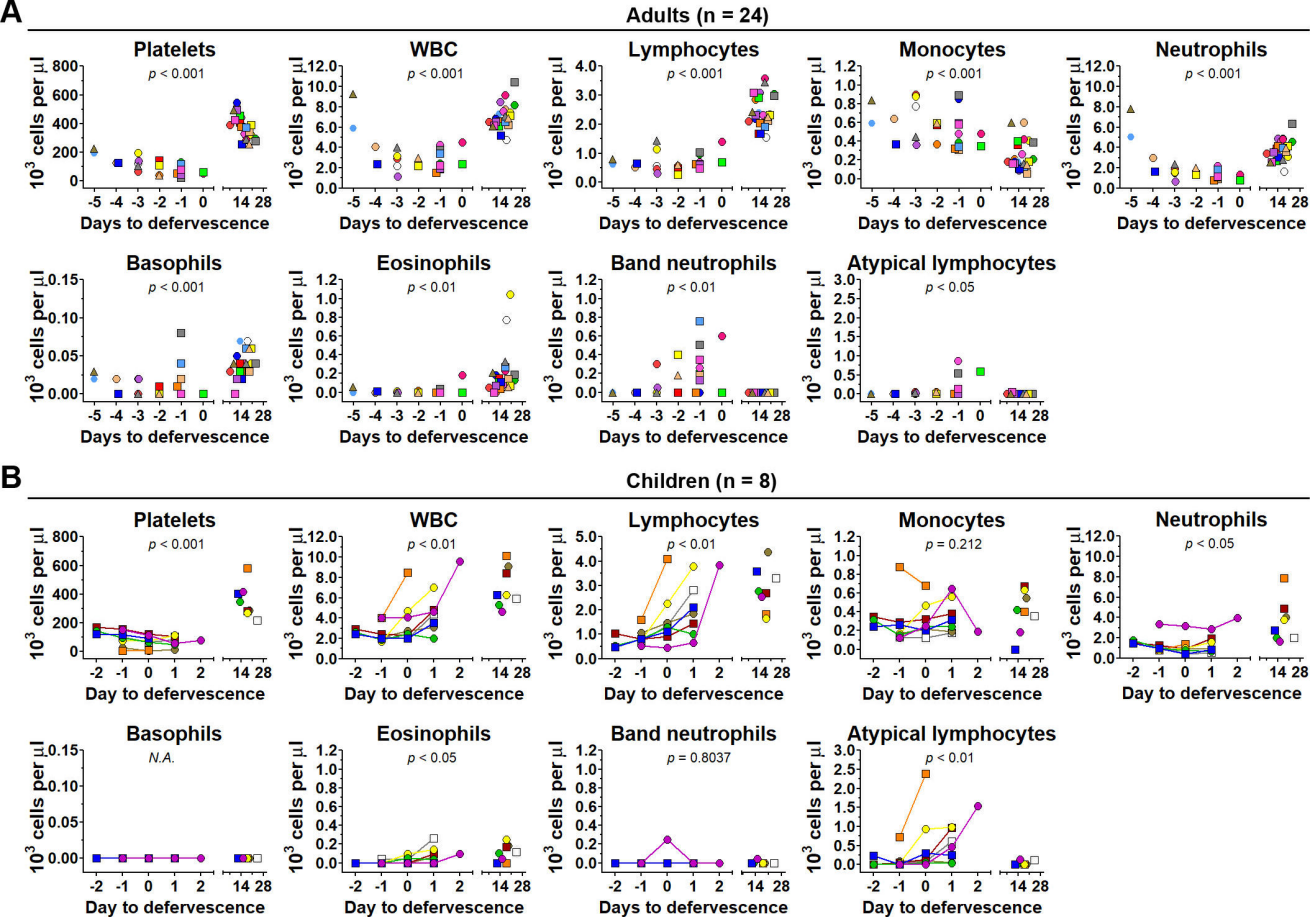
CBC and cell differential count in adult and pediatric dengue patients. Total cell counts and differential counts of white blood cell (WBC) populations (lymphocytes, monocytes, neutrophils, eosinophils, basophils, atypical lymphocytes, band neutrophils) and platelets were determined by standard CBC for whole-blood samples collected from adult (**A**) and pediatric (**B**) dengue patients at the same time points as in Fig. 3. Circles and squares represent DF and DHF patients, respectively. Data are presented as dot plots with unique colored symbols representing individual patients. A Student’s *t*-test was used to analyze the differences in cell numbers between acute (days −2 to 2) and convalescent (days 12 to 24) specimens. *P* values are labeled on each graph.

Given our evidence of DENV infection in various leukocyte populations, we next investigated whether these infections correlate with changes in circulating cell counts during acute dengue illness. Complete blood counts (CBCs) and differential analyses revealed significant alterations in both adult and pediatric patients during the febrile phase compared to convalescence (Fig. 4A and B). Notably, we observed a significant decrease in platelets and total WBC counts (*P* < 0.001), consistent with previous reports of thrombocytopenia and leukopenia in dengue patients (42–44).

In both adults and children, the decrease in WBC counts was primarily driven by a reduction in neutrophils, the most abundant granulocyte type, accompanied by an increase in immature band neutrophils (more pronounced in adults). This suggests increased neutrophil turnover and activation (45), consistent with our *in vitro* and *ex vivo* observations of DENV infection in CD66^+^ granulocytes, which are predominantly neutrophils (Fig. 1 to 3). Notably, eosinophils were dramatically reduced during the acute phase in both adult and pediatric patients (Fig. 4A and B). While basophils were also reduced in adults, this could not be assessed in children due to undetectable baseline levels (Fig. 4B). Given that both eosinophils and basophils express CD66, the reduction of these granulocyte subsets could also be attributed, at least in part, to DENV infection, as observed in our *in vitro* model.

In line with some previous studies (46–48), we observed a significant increase in monocytes in adult patients during the acute phase (Fig. 4A), while this was not clearly observed in pediatric patients. This may be due to the limited number of pediatric patients analyzed in the study. This increase in monocytes in adults suggests their bone marrow expansion and reflecting their mobilization to the circulation, potentially supporting ongoing DENV replication in the blood (Fig. 1 to 3) (49, 50). Lymphocytes were also reduced during acute dengue in both adults and children, coinciding with an increase in atypical lymphocytes, often identified as activated memory B cells (51). This may indicate B-cell activation and differentiation in response to DENV infection and may be a consequence of direct infection of B cells, as observed in our *in vitro* whole-blood infection model (Fig. 1 and 2) and *ex vivo* whole-blood analyses from our study (Fig. 3) and previous reports (52, 53).

The observed alterations in circulating blood cell populations during acute dengue likely result from a complex interplay of factors, including direct viral infection, cellular recruitment to infection sites, and immune-mediated responses. These processes can lead to various outcomes, such as cell death, dysfunction, activation, differentiation, or suppression. While we did not explore these mechanisms in this study, further investigation is needed to elucidate the precise mechanisms underlying these hematological changes and their contribution to dengue pathogenesis.

## DISCUSSION

In this study, we established an *in vitro* whole-blood model using hirudinized blood to study DENV infection, a method previously successful in bacterial infection research. This model allowed us to assess the susceptibility of individual cell types to DENV2 strain 16681 infection and confirm productive viral replication and progeny production. Consistent with prior studies using dengue patient blood samples (52), our findings demonstrate that all major peripheral blood mononuclear cell (PBMC) populations are susceptible to DENV infection, including monocytes, B cells, T cells, and NK cells, albeit with varying degrees. Monocytes remain the most susceptible target cells, aligning with previous research (54, 55). We also observed direct DENV infection of B cells, independent of antibody-dependent enhancement (ADE), corroborating findings in a Cambodian pediatric cohort (53). In contrast, T cells showed very low degrees of viral susceptibility and progeny production, consistent with previous studies demonstrating direct infection only at a very high viral dose (up to 100 multiplicity of infection) (56).

Our *in vitro* data also confirm previous findings about platelet and DENV infection: platelets can bind efficiently to DENV (Fig. 1). This is not surprising, as circulating platelets have been shown to bind to several viruses, including herpes simplex virus (57), vaccinia virus (58), human immunodeficiency virus 1 (HIV-1) (59), hepatitis C virus (60), echovirus 1 (61), and hantavirus (62). However, our results and previous studies (35, 36) suggest that although DENV can be endocytosed and replicated in the cytoplasm of platelets, it cannot generate virus progeny. Failure of virus progeny production in platelets might be due to the scattered structures of the Golgi apparatus and dispersed Golgi sub-compartments (63), which could support virus replication but not virus assembly.

While our model confirms monocytes and B cells as primary targets, consistent with previous studies (52–55), it also revealed unexpected findings regarding B-cell infection. Although B cells were highly susceptible to DENV binding and infection, as demonstrated by significant levels of surface E, intracellular NS1 and NS3, and cell-associated viral RNA upon incubation with DENV (Fig. 1 and 2), they predominantly produced non-infectious virions. Virus progeny generated by infected B cells exhibited a lower specific infectivity than that of NK cells (Fig. 2G), which are much less susceptible to infection (Fig. 1 and 2E). This observation suggests that B cells may play a more complex role in DENV infection than previously thought, potentially serving as a site of viral sequestration or contributing to immune dysregulation rather than solely supporting viral replication.

Our model also revealed a previously unrecognized susceptibility of granulocytes to DENV infection. Granulocytes not only harbored high levels of viral RNA but also efficiently produced infectious virus progeny, suggesting their potential contribution to viral dissemination and pathogenesis. This finding aligns with our *ex vivo* analysis of patient blood samples, where we observed a significant decrease in circulating neutrophils, the most abundant granulocyte type, during acute dengue infection, accompanied by an increase in immature band neutrophils (Fig. 4). This suggests increased neutrophil turnover and activation, potentially linked to direct DENV infection. While we did not directly demonstrate the production of viable virus particles from isolated neutrophils without BHK cell co-cultivation, the presence of NS1 and NS3 strongly suggests active viral replication withing these cells. Future studies utilizing advanced techniques, such as single-cell RNA sequencing or direct visualization of viral particles within neutrophils, could provide more definitive evidence of productive infection. Furthermore, this model could allow the investigation of the impact of DENV infection on neutrophil function and survival, including neutrophil extracellular trap (NET) formation, inflammatory cytokine secretion, granule content release, and reactive oxygen species production, within the context of whole blood. Such studies would help clarify whether infected neutrophils contribute to disease severity by acting as a significant reservoir for DENV replication, promoting inflammation, or impairing host defense mechanisms.

Furthermore, our *ex vivo* analysis of patient blood samples from both adult and pediatric patients confirmed infection in granulocytes, monocytes, and B cells, and to a lesser extent NK and T cells, across all four DENV serotypes. This validates the clinical relevance of our model and highlights granulocytes as a potential target for DENV in humans, suggesting that DENV tropisms in the circulation may not differ significantly among serotypes. However, future studies incorporating other serotypes and clinical isolates in the whole-blood model are warranted.

The observed variability in antigen-positive cell percentage across different cell types and patients, independent of viremia levels, underscores the complexity of DENV tropism *in vivo*. This suggests that DENV cellular tropism is a multifaceted process influenced by factors beyond viral load alone, such as differential cellular permissiveness to DENV serotypes, the host’s immune status (influenced by prior DENV exposure), and the dynamic interplay between viral replication and host responses over the course of infection. These complex interactions may underlie the observed alterations in circulating blood cell populations during acute dengue, including thrombocytopenia, leukopenia, neutropenia, monocytosis, and increased atypical lymphocytes and band neutrophils. Future longitudinal studies, particularly controlled human challenge studies that enable early sample collection, will be essential to dissect these complex interactions. Understanding the interplay between these factors could reveal novel therapeutic targets aimed at modulating DENV cellular tropism and mitigating the hematological manifestations of dengue.

In summary, our hirudinized whole-blood model offers a valuable tool for investigating DENV pathogenesis by effectively recapitulating early infection dynamics and preserving crucial immune interactions. This model has revealed the susceptibility of granulocytes to DENV infection, highlighting their potential role in disease progression. Future studies utilizing this platform can further investigate the impact of DENV infection on various cell types, explore diverse immune responses, and investigate the effects of sequential infections, ultimately contributing to the development of novel therapeutic strategies targeting these interactions.

## MATERIALS AND METHODS

### Antibodies and reagents

Anti-DENV envelope (E) antibody (clone 4G2) was provided by the Armed Forces Research Institute of Medical Sciences (AFRIMS) (64). Anti-DENV nonstructural protein NS1 (clone 1F11) and anti-NS3 (clone E1D8) antibodies were kindly provided by Chunya Puttikhunt (65) and Eva Harris (66), respectively. Anti-DENV NS5 polyclonal antibody (GTX103350) was purchased from GeneTex (CA, USA). Phycoerythrin (PE)-conjugated streptavidin, peridinin–chlorophyll–protein complex (PerCP)-conjugated mouse anti-human CD14 (clone HCD14), allophycocyanin/cyanine 7 (APC/Cy7)-conjugated mouse anti-human CD19 (clone HIB19), Brilliant Violet 510 (BV510)-conjugated mouse anti-human CD3 (clone OKT3), and Zombie Yellow fixable viability kit were purchased from BioLegend (CA, USA). Phycoerythrin/cyanine 7 (PE/Cy7)-conjugated mouse anti-human CD56 (clone B159), Brilliant Violet 421 (BV421)-conjugated mouse anti-human CD66 (clone B1.1), and permeabilizing buffer (Perm2) were purchased from BD Biosciences (NJ, USA). Hirudin was purchased from US Biological (MA, USA). Allophycocyanin (APC) and R-phycoerythrin (R-PE) conjugation kits were purchased from Innova Biosciences (Cambridge, UK). Hirudin and EDTA were purchased from US Biological (MA, USA). APC and R-PE conjugation kits were purchased from Innova Biosciences (Cambridge, UK). Anti-human C3b antibody medium was purchased from Quidel (CA, USA). FITC-conjugated rabbit anti-mouse IgG was purchased from DAKO (CA, USA). RPMI 1640 medium was purchased from Thermo Fisher Scientific (MA, USA).

### Human specimens

This study used EDTA–blood specimens from the following sources: specimens from three DENV non-immune adults (no neutralizing antibodies against DENV2, data not shown) were used to establish the *in vitro* whole-blood model of DENV infection (Protocol Number: 635/2557). Specimens were collected from 24 adult dengue patients admitted to Siriraj Hospital (November 2015 to December 2017) at both acute and convalescent phases. Specimens were collected from eight pediatric dengue patients enrolled in Khon Kaen and Songkhla Hospitals (February 2018 to September 2019) daily during the acute phase and at the convalescent phase (1–4 weeks after fever onset). Specimens from six healthy adults were used as negative controls (Protocol Number: 635/2557). DENV infection was confirmed in all patient specimens using DENV IgG/IgM-capture ELISA (67), conventional nested RT-PCR for serotyping (68), and NS1 ELISA (69). Patient severity was classified according to the World Health Organization 1997 guidelines (42) (Table 1).

**TABLE 1:** Demographic of dengue patients

Characteristic	Adults (*n* = 24)	Children (*n* = 8)
Age (mean ± SD, years)	29 ± 12	13 ± 1
Dengue serology (primary:secondary)	0:24	2:6
Disease severity (DF:DHF)	16:8	4:4
Specimen collection date (days to defervescence, range)		
Acute	Days −5 to 0	Days −2 to 2
Convalescence	Days 7 to 23	Days 12 to 24
Dengue serotypes (*n*)		
DENV 1	4	5
DENV 2	10	4
DENV 3	1	1
DENV 4	9	0

### Cells and viruses

Baby hamster kidney (BHK) and Vero cell lines were cultured as described previously (70) for use in virus propagation and titration assays, respectively. DENV serotype 2 strain 16681 (DENV2) was propagated in C6/36 cells, then concentrated by filtration (10-kDa cutoff, Amicon) and partially purified by centrifugation in a 24% (wt/wt) sucrose buffer (32). The purified DENV pellet was resuspended in RPMI 1640 medium, and viral titers were determined using both FFA (71) and real-time quantitative RT-PCR (72). The virus stock was stored at −70°C until use.

### *In vitro* DENV infection in a whole-blood culture model

One milliliter of whole blood from DENV non-immune healthy volunteers was immediately added to tubes containing recombinant hirudin (final concentration of 50 µg/mL) to preserve complement activity. To assess complement function, hirudinized plasma was incubated with yeast cells (strain Y187, provided by Dr. Thawornchai Limjindaporn, Faculty of Medicine Siriraj Hospital) for 30 min at room temperature. Levels of the complement fragment C3b deposited on the yeast cell surface were determined by flow cytometry using an anti-human C3b antibody followed by FITC-conjugated rabbit anti-mouse IgG. EDTA (10 mM) was used as a negative control to inhibit complement activation. For the DENV infection model, whole blood in hirudin-containing tubes was gently mixed by inversion and then incubated with partially purified DENV2 (10⁷ or 10⁸ genome copies) for 2 and 18 h at 37°C with rotation. Infectious DENV titers in whole blood were determined by FFA (71).

### Immunofluorescent staining and flow cytometry

At 2 and 18 h post *in vitro* DENV infection, whole-blood samples were collected for analysis of platelets (2-µL samples) and white blood cells (WBC; 50-µL samples). For WBC preparation, erythrocytes in 50 µL of whole blood were lysed with 1× RBC lysing buffer (150 mM ammonium chloride, 10 mM potassium bicarbonate, 1.25 mM EDTA) for 10 min at room temperature. Cells were then washed with washing buffer (20 mM EDTA in phosphate-buffered saline) and stained with Zombie Yellow for 10 min at room temperature. After washing, cells were stained with antibodies against individual CD markers for 30 min at room temperature. For intracellular staining, CD marker-stained cells were fixed and permeabilized, then stained with PE-conjugated anti-NS1 or APC-conjugated anti-NS3 antibodies. For pediatric specimens, CD marker-stained cells were stained with biotinylated anti-NS1 (clone 1F11) followed by PE-conjugated streptavidin. All cells were washed with washing buffer after staining and analyzed on an LSRFortessa flow cytometer using FACSDiva software (BD Biosciences, CA, USA).

### Cell isolation and DENV-infected cell analysis

After 18 h of DENV infection, platelets and leukocyte populations were isolated from mock- and DENV-infected whole blood. Platelet-rich plasma (PRP) was obtained by centrifugation (200 × *g*, 20°C, 10 min), and platelets were then isolated from PRP (1,500 × *g*, 20°C, 20 min). Isolated platelets were washed three times, and purity was confirmed by CD41 expression (>99% positive). Packed cells remaining after PRP removal were diluted 1:1 with normal saline and centrifuged on Polymorphprep (Axis-Shield, Dundee, UK) to separate leukocytes from RBCs. Leukocytes were washed and stained with CD markers for sorting by FACSAria III (BD Biosciences, NJ, USA). The purity of each isolated leukocyte population was determined by its specific CD marker (CD66 for granulocytes, CD14 for monocytes, CD19 for B cells, CD3 for T cells, and CD56 for NK cells).

To assess intracellular NS5 and NS1 expression, each isolated leukocyte population was fixed, permeabilized, and stained with anti-NS5 (followed by Alexa Fluor 488-conjugated goat anti-rabbit IgG) or anti-NS1 (followed by Alexa Fluor 488-conjugated rabbit anti-mouse IgG). Cells were washed and analyzed using confocal microscopy (Carl Zeiss, Jena, Germany).

To investigate DENV infectivity, isolated platelets and leukocyte populations were divided into two sets (each set contained approximately 75,100 ± 53,800 granulocytes, 32,800 ± 25,200 monocytes, 203,500 ± 137,900 T cells, 29,500 ± 17,100 B cells, 47,700 ± 44,400 NK cells, and 23,500,000 ± 18,100,000 platelets). One set was used for viral genome quantification by TRIZol extraction and real-time quantitative RT-PCR (72). The other set was used in an infectious cell center assay by coculturing each isolated cell population with a BHK cell monolayer in 48-well plates for 2 days (73). Infectious virus titers and viral genome in supernatants were determined using FFA (71) and real-time quantitative RT-PCR (72), respectively. The absence of extracellular virus contamination was confirmed in the final wash buffer of each cell population.

### Statistical analysis

Statistical analyses were performed using GraphPad Prism software (version 5.00). Data distribution normality was determined using the Kolmogorov–Smirnov test. Comparisons between groups were made using either the *t*-test (for normally distributed data) or the Mann–Whitney test (for non-normally distributed data). A *P*-value ≤ 0.05 was considered statistically significant. Data represent results from 3 to 10 independent experiments.

## SUPPLEMENTAL MATERIAL

Supplemental figures mbio.01505-24-s0001.docx Fig. S1 and S2.

ASM does not own the copyrights to Supplemental Material that may be linked to, or accessed through, an article. The authors have granted ASM a non-exclusive, world-wide license to publish the Supplemental Material files. Please contact the corresponding author directly for reuse.
