# Supplementary material for: Whole-blood model reveals granulocytes as key sites of dengue virus propagation, expanding understanding of disease pathogenesis
Source: mBio. 2024 Nov 14;15(12):e01505-24. doi: 10.1128/mbio.01505-24 (PMC11633123; doi:10.1128/mbio.01505-24)
Supplement: Supplemental figures — Fig. S1 and S2. [file mbio.01505-24-s0001.docx]

**Supplemental material**

**Fig S1**

**
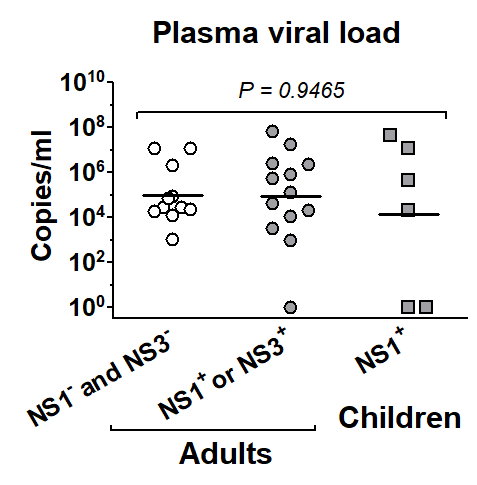
**

**Fig S1. Comparison of** **plasma viral load between patients with and without detectable DENV NS1 or NS3 in white blood cell populations.** Plasma viral load was measured by qRT-PCR in 24 adult and 6 pediatric patients during the acute phase of DENV infection. Adult patients were grouped based on the presence or absence of NS1 and NS3 in any white blood cell (WBC) population. All pediatric patients had detectable NS1 in at least one WBC population. Each symbol represents the plasma viremia level from an individual patient.

**Fig S2**

**
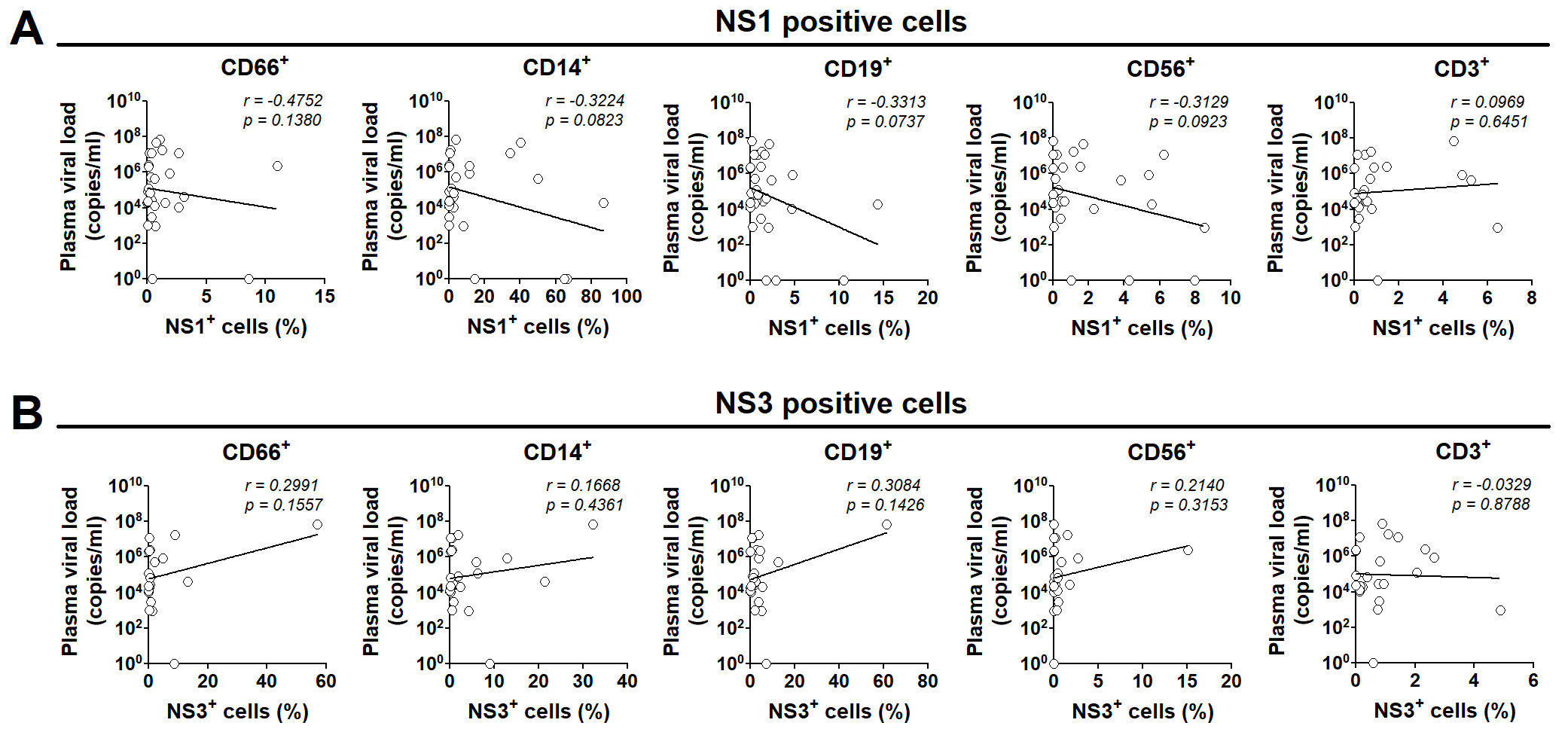
**

**Fig S2. Correlation between plasma viral load and percentage of DENV NS1- or NS3- positive cells in white blood cell populations.** Pearson correlation analysis was conducted to evaluate the relationship between plasma viral load and the percentage of NS1-positive (**A**) or NS3-positive (**B**) cells within each white blood cell population, using data from 24 adult and 6 pediatric dengue patients. One data point for NS1-positive CD66^+^ granulocytes in adults was excluded due to an extremely high percentage of NS1-positive cells (46%) and undetectable plasma viral load, which was considered an outlier. Including this outlier in the analysis yielded a correlation coefficient (*r*) of -0.4466 and *p*-value of 0.0134, while excluding it resulted in an *r* of -0.4752 and a *p*-value of 0.1380, as shown in the figure. For CD3^+^ T cells in pediatric patients, plasma viral load data was only available for one out of two data points. Correlation coefficients (*r*) and *p*-values (*p*) are indicated on each graph.
